# Radiation-Induced Dyspnea in Lung Cancer Patients Treated with Stereotactic Body Radiation Therapy

**DOI:** 10.3390/cancers13153734

**Published:** 2021-07-25

**Authors:** Laura Cella, Serena Monti, Maria Thor, Andreas Rimner, Joseph O. Deasy, Giuseppe Palma

**Affiliations:** 1 Institute of Biostructures and Bioimaging, National Research Council, 80145 Napoli, Italy; serena.monti@ibb.cnr.it; 2 Department of Medical Physics, Memorial Sloan Kettering Cancer Center, New York, NY 10065, USA; thorm@mskcc.org (M.T.); DeasyJ@mskcc.org (J.O.D.); 3 Department of Radiation Oncology, Memorial Sloan Kettering Cancer Center, New York, NY 10065, USA; rimnera@mskcc.org

**Keywords:** stereotactic body radiation therapy, dyspnea, risk factors, NTCP

## Abstract

**Simple Summary:**

Dyspnea is a common symptomatic side-effect of thoracic radiation therapy. The aim of this study is to build a predictive model of any-grade radiation-induced dyspnea within six months after stereotactic body radiation therapy in patients treated for non-small cell lung cancer. The occurrence of pre-treatment chronic obstructive pulmonary disease and higher relative lungs volume receiving more than 15 Gy as well as heart volume were shown to be risk factors for dyspnea. The obtained results encourage further studies on the topic, which could validate the present organ-based findings and explore the voxel-based landscape of radiation dose sensitivity in the development of dyspnea.

**Abstract:**

In this study, we investigated the prognostic factors for radiation-induced dyspnea after hypo-fractionated radiation therapy (RT) in 106 patients treated with Stereotactic Body RT for Non-Small-Cell Lung Cancer (NSCLC). The median prescription dose was 50 Gy (range: 40–54 Gy), delivered in a median of four fractions (range: 3–12). Dyspnea within six months after SBRT was scored according to CTCAE v.4.0. Biologically Effective Dose (α/β = 3 Gy) volume histograms for lungs and heart were extracted. Dosimetric parameters along with patient-specific and treatment-related factors were analyzed, multivariable logistic regression method with Leave-One-Out (LOO) internal validation applied. Model performance was evaluated by the area under the receiver operating characteristic (ROC) curve (AUC) and calibration plot parameters. Fifty-seven patients (53.8%) out of 106 developed dyspnea of any grade after SBRT (25/57 grade ≥ 2 cases). A three-variable predictive model including patient comorbidity (COPD), heart volume and the relative lungs volume receiving more than 15 Gy was selected. The model displays an encouraging performance given by a training ROC-AUC = 0.71 [95%CI 0.61–0.80] and a LOO-ROC-AUC = 0.64 [95%CI 0.53–0.74]. Further modeling efforts are needed for dyspnea prediction in hypo-fractionated treatments in order to identify patients at high risk for developing lung toxicity more accurately.

## 1. Introduction

Thoracic radiation therapy (RT) is often associated with the risk of developing acute or late radiation-induced lung damage (RILD), which may lead to dyspnea, lung fibrosis, and impaired quality of life (QoL) [1,2,3]. Among the high conformal RT modalities [4], stereotactic body RT (SBRT) is currently considered the treatment of choice for inoperable early-stage non-small cell lung cancer (NSCLC) and an alternative treatment option for pulmonary metastases [5]. The increasing use of hypo-fractionated treatments delivered by SBRT implies high doses per fraction, larger than 10 Gy and up to 20–30 Gy [6,7], claiming the need for dedicated normal tissue complicated probability (NTCP) models for RILD [4]. A range of clinical and dosimetric parameters have previously been shown to be predictive of RILD after lung tumors hypo-fractionated RT in several studies [8,9,10,11,12,13,14] though producing conflicting results [15].

Furthermore, with SBRT being increasingly used in NSCLC elderly patients with significant comorbidities [16], there is growing attention to those symptoms impacting on QoL after treatment, including symptoms related to lung disease as dyspnea [17,18,19]. For this subset of frail patients, it is indeed important to balance patient and treatment factors in selecting the optimal patient for SBRT [5,20].

In the present study, we analyzed the incidence of radiation-induced dyspnea in a cohort of patients treated for NSCLC with SBRT and we investigated the clinical and dosimetric prognostic factors for the development of a robust toxicity prediction model.

## 2. Materials and Methods

### 2.1. Subsection

The study involved 106 patients treated for NSCLC with SBRT at Memorial Sloan Kettering Cancer Center between 2012 and 2016 (IRB #16-142) for whom clinical and dosimetric information were available for the present retrospective analysis. Patient and treatment characteristics are shown in Table 1. Details of treatment characteristics are published in previous papers [21,22]. Briefly, the gross tumor volume (GTV) was contoured and an internal target volume (ITV) was generated from the phase-averaged respiratory-correlated CT scan. The clinical target volume (CTV) included the ITV with 2–3 mm uniform expansion for including microscopic disease extension, and the planning target volume (PTV) included the CTV plus a uniform expansion of 5 mm. Dose was prescribed to the 100% isodose line and delivered with coplanar Intensity Modulated RT beams using the Eclipse treatment planning system (Eclipse v.13, Varian Medical Systems, Palo Alto, CA, USA).

The median PTV prescription dose was 50 Gy (range: [40, 54 Gy]), delivered in a median of 4 fractions (range: 3–12). The associated median Biological Effective Dose (BED, α/β = 10 Gy) was 105.6 Gy (range: [67.2, 151.2] Gy).

Patients were clinically evaluated during RT, approximately 1 month after completion of SBRT and every 3–6 months thereafter. From routine follow up visits based on a retrospective review of the note, for each patient, dyspnea within 6 months after the beginning of SBRT was scored according to the National Cancer Institute’s Common Terminology Criteria for Adverse Events v.4.0 [23] into the following groups:

Grade 1: Shortness of breath with moderate exertion

Grade 2: Shortness of breath with minimal exertion, limiting instrumental Activities of Daily Living (ADL)

Grade 3: Shortness of breath at rest, limiting self-care ADL

Grade 4: Life-threatening consequences, urgent intervention indicated

Grade 5: Death.

### 2.2. Dosimetric and Statistical Analysis

For each patient, a voxelwise conversion of physical doses to BED was performed using an α/β = 3 Gy, and BED volume histograms (BED-VHs) for normal lungs (with the exclusion of the GTV [24]) and heart were computed; the relative volumes receiving ≥ x Gy (V_x_) in steps of 5 Gy were extracted along with the near maximum dose (D_2%_) and the mean dose (D_mean_) to the considered organs.

The extracted pulmonary and cardiac dosimetric parameters, as well as patient-specific and treatment-related variables, were analyzed by univariable statistical methods for patients grouped according to dyspnea occurrence. Pearson’s χ^2^-test or Fisher’s exact test were adopted for categorical variables, while Mann–Whitney U-test was used for continuous variables.

Average relative BED-VHs grouped according to the toxicity endpoint were compared at each dose point by a two tailed *t*-test. A significance α-level of 0.05 corrected by the Holm–Šidák method for multiple comparisons was adopted [25].

To assess the possible effect of dosimetric and clinical factors on dyspnea, the multivariable stepwise logistic regression method for toxicity risk modeling was applied [26,27,28,29]. We performed a variable preselection performed via a univariable analysis in order to avoid overfitting. Therefore, in the multivariable analysis (MVA), we included only the variables highly correlated with dyspnea (i.e., *p* < 0.1 at the univariable analysis) and that were not collinear (correlation |R_s_| < 0.75) with variables showing a higher correlation with the toxicity outcome. The Leave-One-Out (LOO) method was applied to the whole statistical workflow to cross-validate the model [30,31]. Model performance was quantified by the area under the receiver operating characteristic (ROC) curve (AUC) and Brier score. The agreement between observed outcome and LOO prediction were assessed by calibration plots.

## 3. Results and Discussion

**Figure 1:**
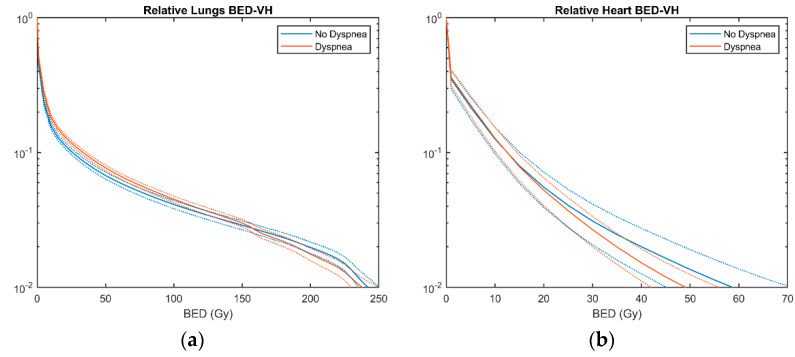
Average Biologically Effective Dose (BED)-Volume Histograms ± SEM (Standard Error of the mean) of patients developing any grade dyspnea and who did not.

Fifty-seven patients (53.8%) out of 106 developed dyspnea of any grade within 6 months after SBRT. Out of the 57 dyspnea cases, 17 (30%) were scored as grade 2 dyspnea and 8 cases were scored as grade 3 (14%). There were no cases of grade 4 and 5 toxicity. The incidence here described is consistent with the heterogeneous data reported in the recent literature on dyspnea incidence after SBRT, which ranges from the relatively low value of 17% [32] to 36% [33] and 66% [34]. The rate of dyspnea occurrence, as well as the distribution of dyspnea grades found in the analyzed cohort, provides support for a relative low rate of severe radiation-induced lung morbidity after SBRT [35]. Thanks to small tumor size combined with small safety margins, the amount of normal lung tissue exposed to hypo-fractionated doses is indeed limited, as we can observe from the relative BED-VH of the lungs shown in Figure 1a. Similarly, an even more limited heart exposure can be observed in Figure 1b.

As for dosimetric parameters, the lung BED-VH metrics in the range of [1, 50] Gy were significantly correlated with dyspnea (Figure 1a). No significant difference was found instead in heart BED parameters between patients developing dyspnea and those who did not.

Besides dosimetric parameters, patient-related factors were found to be associated with the risk of developing RILD. At the univariable analysis for patients grouped according to any grade dyspnea, chronic obstructive pulmonary disease (COPD) (*p* = 0.007), and heart volume (*p* = 0.05) were the only clinical and pre-treatment disease variables significantly correlated with dyspnea.

**Figure 2:**
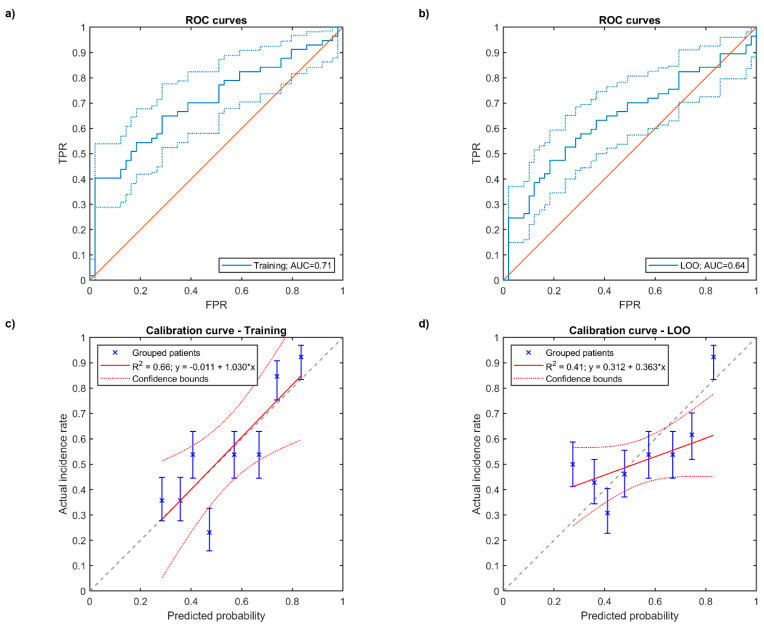
Training (**a**) and cross-validated (**b**) ROC curves of multivariable logistic regression model (FPR: False Positive Rate, TPR: True Positive Rate), training (**c**) and cross-validated (**d**) calibration plots of logistic model. In (**c**,**d**) the error bars for the reported values represent the 68% confidence intervals.

After the variable selection procedure, the MVA resulted in a three-variable model, including the relative lungs volume receiving more than 15 Gy (V_15Gy|α/β=3_), COPD and heart volume (Table 2). The obtained logistic model displayed an acceptable performance given by a training ROC-AUC of 0.71 and by a nearly ideal calibration curve (Figure 2a,c, Table 2). LOO cross-validation confirmed an encouraging ROC-AUC value of 0.64, though a poor result of the calibration test was displayed (Figure 2b,d, Table 2).

The pre-treatment COPD, one of the most frequent causes of surgical inoperability for NSCLC patients [36], has been previously reported as a risk factor for RILD, both after standard fractionated RT [37] and SBRT [38,39]. Although COPD has not been considered as a contraindication for SBRT in early-stage lung cancer patients [39], the understanding of its impact on post-treatment dyspnea and its inclusion in a multivariable NTCP model may aid individualized risk–benefit analysis and plan optimization.

Interestingly, the present study establishes the statistical importance of the heart volume size in the risk prediction of lung toxicity. The dose to the cardiac structures has been shown to influence pulmonary toxicity in both preclinical [40,41] and clinical studies [2,42,43]. Probably, due to the small portion of irradiated cardiac tissue related to SBRT modality, here, we propose an NTCP model that includes the volume size of the heart as a significant prognostic factor for the development of dyspnea with an increasing risk for larger volumes. Consistently with this result, a pronounced “volume effect” has been observed in irradiated rat hearts [44].

As for lung dosimetric parameters, most studies, using the dose-volume histogram derived from physical dose and then regardless of fractionation, found the mean lung dose or the lung V_20Gy_ as predictors of symptomatic RILD after lung SBRT, although better correlation has been reported for volumes irradiated at doses lower than 20 Gy, such as V_5Gy_ and V_13Gy_ [15].

In the present analysis, in order to account for different fractionation regimens, the BED-VH metrics were extracted and the V_15Gy|α/β=3_ resulted to be an independent dose metric predictor for dyspnea, an important symptomatic endpoint for assessment of RILD [45]. Notably, the use of the adopted BED model is not universally accepted, especially when large fraction sizes are considered. However, we believe it is fundamental to account for the different fractionation schemes, which at least in normal tissues do not involve dose levels as high as in the tumor region.

We are aware that an intrinsic limit of our retrospective analysis is the lack of data on pre-RT patients’ dyspnea. For this reason, we included the pre-RT COPD status as a surrogate for missing data on pre-RT dyspnea. Nonetheless, we still believe that the obtained model, once validated in larger cohorts of patients, paves the way for risk prediction in order to move toward a personalized treatment plan optimization in SBRT. Moreover, we highlight the importance of including in future studies more objective measures of lung damage, such as the pulmonary function test.

The study we performed points out the importance of collecting larger cohorts of SBRT lung cancer patients that might support the data size requirements of more sophisticated statistical methods [46,47,48,49,50]. In particular, several studies highlighted the significant spatial variations of radio-sensitivity in heart and lung subregions [51,52,53,54]. We suggest that voxel-based analyses of dosimetric findings related to radiation-induced dyspnea could shed an even clearer light on the pathophysiological pathways of RT-related toxicity, possibly solving the apparent inconsistency in organ-based dose predictors.

## 4. Conclusions

In the analyzed cohort of NSCLC patients treated with SBRT, a low frequency of severe dyspnea was observed. Twenty-four percent of patients developed dyspnea of grade ≥2 within six months after RT.

Patients’ comorbidity (COPD) and individual patient characteristic (heart volume) along with V_15Gy|α/β=3_ resulted in being independent prognostic factors for dyspnea of any grade after lung SBRT. Further modeling efforts are needed for RILD prediction in hypo-fractionated treatments in order to identify more accurately patients at high risk for developing lung toxicity impacting of their QoL. The standardized use of BED-VH could help future data pooled analysis.

## Figures and Tables

**Table 1:** Patient and treatment characteristics.

Characteristics	*n* = 106
**Continuous variables**	**Median (Range)**
Age at RT (yr.)	75 (32–93)
Lung-GTV Volume (cm^3^)	3013 (1537–7644)
GTV (cm^3^)	6.4 (0.3–162.9)
Heart Volume (cm^3^)	682 (340–1316)
KPS baseline (%)	90 (60–100)
**Categorical variables**	***n* (%)**
Gender	
Male	45 (43)
Female	61 (57)
**Histology**	
Adenocarcinoma	82 (77)
Squamous Cell Carcinoma	16 (15)
Unknown	8 (8)
**Tumor position**	
Right lung	66 (62)
Left lung	40 (38)
Upper lobe	61 (58)
Middle lobe	4 (4)
Lower lobe	41 (39)
**COPD**	
No	61 (58)
Yes	45 (42)
**Smoking**	
Never	15 (14)
Former	80 (76)
Current	11 (10)
**Fractionation schedule**	
18 Gy × 3 fx	24 (23)
12 Gy × 4 fx	38 (36)
10 Gy × 5 fx	33 (31)
9 Gy × 5 fx	7 (7)
8 Gy × 5 fx	4 (4)
**Dyspnea within 6 months**	
Grade 0	50 (47)
Grade 1	31 (29)
Grade 2	17 (16)
Grade 3	8 (8)

Abbreviations: KPS = Karnofsky Performance Score; fx = fraction, COPD = Chronic Obstructive Pulmonary disease.

**Table 2:** Multivariable logistic regression model coefficient and model performance for any grade dyspnea, 95% confidence intervals are in brackets.

Model Variables	Coefficient	SE	*p*
COPD	1.02	0.43	0.02
Lungs V_15Gy|α/β=3_	9.3	4.6	0.04
Heart Volume (cc)	0.0021	0.0011	0.06
constant	−3.09	1.09	0.004
AUC	0.71 (0.61–0.80)		
CV-AUC	0.64 (0.53–0.74)		
Brier score	0.215		
CV-Brier score	0.236		
Calibration intercept	−0.01	0.17	
Calibration slope	1.03	0.30	
CV-Calibration intercept	0.31	0.11	
CV-Calibration slope	0.36	0.20	

Abbreviations: V_x_: Percentage volume receiving at least x Gy; COPD = Chronic Obstructive Pulmonary disease; SE = Standard Error; CV = Cross Validation.

## Data Availability

The data presented in this study are not publicly available due to restrictions in the Material Transfer Agreements.
